# A Hospital in the Making
*By B. Burford Rawlings. (Pitman. 5s. net.)


**Published:** 1913-03-15

**Authors:** 


### "A HOSPITAL IN THE MAKING." *

### MR. B. BURFORD RAWLINGS' HISTORY OF THE NATIONAL HOSPITAL.

At the present time, when public opinion generally has become increasingly focussed on the voluntary hospitals of this country, it is well that the public should be informed at first hand of the history and growth of one of these institutions by a writer who is really qualified to express an opinion. The fact that the National Hospital for the Paralysed and Epileptic belongs to the small class of special hospitals, whose needs may not appeal to so wide a circle as our large general hospitals, serves to enhance the value of the book as a record of a notable achievement, and must deeply impress any reflective mind with the characteristic attitude of the British public at large towards philanthropic objects.

The hitherto instinctive distrust of the Englishman for State aided or supported institutions, the remarkable combination of lay administration with highly skilled professional service, the acceptance, or rather the disinterested tolerance, by the State of independent philanthropic effort, all these factors in our national life are brought into prominence in a remarkable degree by a study of Mr. Rawlings' delightful book. Permeating the entire work is an atmosphere of ungrudging personal service and ennobling effort, which, in these later days of self-advancement and advertisement, especially in the field of so-called charitable work, is so often conspicuous by its absence. There can be no doubt that it was never the intention of Miss Chandler or her brother, to whose early efforts the successful establishment of the hospital was due, that lay control should give place to staff control, for, to use Mr. Chandler's own words, the institution was to be "no doctor's shop," and yet all will remember the great controversy which culminated with Sir Edward Fry's Committee of Inquiry in 1901, in the result of which the claims of the staff to representation were more than amply recognised. Possessed of the most intimate knowledge of the special needs and requirements of the hospital, the mainstay of its financial resources, and for thirty-five years the jealous guardian of its reputation and welfare, Mr. Rawlings describes his severance with the institution in these touching words: "With the incoming of the new Board my office ceased automatically, and having enclosed my keys in a packet addressed to the newly-elected president, the Earl of Dudley, I went forth. As a not unhappy chance befell, I encountered nobody. I was the solitary occupant of the footway, and glad of the solitude, even though it meant no kindly anæstheticism of solace to dull the pain of severance from a prized task and of the putting off of harness, which, if it gripped often, never had galled.

"But no man has lost all so long as memory is indulgent . . . In his illusions, perhaps, he is happiest, for life would be poorer for any one of us without them, and hope herself would lose her best and kindliest quality, if she did not from time to time, in perfect good faith, misread the future."

### Patients' Payments in the Seventies.

One of the most interesting topics dealt with by the author is that concerning the question of patients' payments. He recalls the fact that in the early seventies none of the great hospitals admitted contributing patients, and deplores the consequent denial of self-help to hospital patients as a class. "That the persons most suitable to become patients of hospitals are those not wholly destitute is a truism much too frequently overlooked, and when inquiry has made plain that an applicant is poor enough to be eligible, what can be more unreasonable than that an indigent institution should refuse such assistance as he is capable of giving, or that the patient should be deprived of his privilege of self-help?" With these sentiments we entirely concur, and it is devoutly to be hoped that the day is not far distant when the voluntary hospitals as a class will realise the importance of furnishing an adequate proportion of beds for those inmates who are prepared, and rightly so, to share in the expense of the treatment which they seek.

### A Masterpiece of Appeal-writing.

In view of the precarious nature of the sources of revenue of most of our voluntary hospitals, Mr. Rawlings' description of the manner in which he commenced the difficult task of raising well-nigh £100,000 for the re-building of the hospital in 1882 will repay perusal by those at present charged with the duty of raising the

* By B. Burford Rawlings. (Pitman. 5s. net.)

necessary funds for their own institutions. But the author's masterpiece in appeal writing was achieved in "Echoes of Many Voices," every sentence of which was a literal transcript from the published utterance of an eminent advocate on behalf of the "Hospital in Queen Square." Mr. Rawlings rightly attributes the success of his method of appeal to the fact that no statement was made and no argument applied without the warrant of high independent testimony—a salutary rule which is not always so strictly observed by hospital and other charitable authorities as could be desired.

The book is so full of good things that within the limits available it is almost impossible to do the author justice, but this review would not be complete if Mr. Rawlings' conception of the qualities which combine to make a good hospital manager were omitted. We make no apology for quoting this passage in full.

The Ideal Manager Described.

"The most capable administrator would prove a dangerous doctor, an incompetent nurse, and a death-dealing dispenser, while the amplitude of his knowledge enables him to realise the limits beyond which his ignorance is profound. A manager who aims at efficiency must be many-sided. He needs to be a good man of business, a capable correspondent, an accountant, the possessor of some literary power, a trustworthy adviser, a reliable interpreter and upholder of rules, a supporter of the authority vested in other people, and capable of exercising his own; if not a speaker, competent to supply speeches for someone else's utterance, a man resourceful in emergencies and able to solve puzzles by intuition, to check expenditure of all kinds, a successful beggar, an enthusiast never numbed by the cold indifference which often awaits his most cherished schemes, patient under opposition and disappointment, yet always persistent, a realisation and embodiment in his own person of the qualities of the famous policeman who captured a burglar, and when asked how he did it single-handed replied, 'I surrounded the house.'"

The perfect good taste and literary merit of the book, together with the absorbing narrative it unfolds, must commend it to all who are genuinely interested in the voluntary hospital system of this country as it exists to-day. We have always regretted the loss to the voluntary hospitals of the able pen Mr. Burford Rawlings is so capable of using effectively. May we venture to hope that the publication of this book and its deserved success may encourage Mr. Rawlings to devote himself to literary work dealing mainly with hospital subjects? We can at any rate promise him a hearty welcome in this field.

